# Exploring client and clinician experiences of cognitive behavioural therapy for depersonalisation-derealisation disorder (CBT-f-DDD)

**DOI:** 10.1186/s12888-026-08052-7

**Published:** 2026-05-05

**Authors:** Cheuk Lon Malcolm Wong, Elaine C.M. Hunter, Toby Newson, Nicola Dalrymple, Joe Perkins, Nicola Morant

**Affiliations:** 1 Division of Psychiatry, University College London, London, UK; 2 Department of Psychology, Royal Holloway University of London, Egham, UK; 3 Unreal Charity, Bristol, UK

**Keywords:** Cognitive behavioural therapy, Depersonalisation, Derealisation, Dissociation, Acceptability, Feasibility, Qualitative, Treatment experience

## Abstract

**Background:**

Depersonalisation-derealisation disorder (DDD) is an under-recognised condition, involving a sense of detachment from one’s internal processes and environment. Cognitive behavioural therapy for DDD (CBT-f-DDD) has shown promising quantitative outcomes in previous research. However, there is a lack of research examining the inner workings of CBT-f-DDD and how the treatment is experienced. This qualitative study explored the experiences and perspectives of clients who received CBT-f-DDD, and the clinicians who delivered this, to better understand acceptability, feasibility of applying CBT-f-DDD more widely in the NHS, and therapeutic processes that potentially contributed to outcomes.

**Methods:**

The study was embedded within a CBT-f-DDD feasibility randomised controlled trial in the UK. Participants consisted of clients who completed therapy and clinicians recruited from the treatment arm of the trial. Semi-structured interviews were conducted with seven clients and seven therapists after therapy. Data were analysed using codebook thematic analysis.

**Results:**

Findings clustered into four broad domains are presented: “reception of therapy”, “possible therapeutic processes as drivers for change”, “improving the therapy”, and “treatment implementation in the NHS”. Clients expressed appreciation for being offered the opportunity to engage in CBT-f-DDD. CBT was considered an appropriate approach to treating DDD and treatment completers saw various therapeutic benefits, including a decrease in symptoms, improved relationship with DDD and higher confidence in managing dissociation. A few clients shared that interventions were not sufficiently targeted. Challenges of accommodating comorbid mental health difficulties within the CBT-f-DDD model were described by a few clinicians. Clinicians viewed CBT-f-DDD as feasible for delivery in the NHS. However, establishing effective treatment pathways and facilitating appropriate training and supervision for clinicians were considered important prerequisites. Building a strong therapeutic rapport, constructing a shared understanding of DDD and the reframing of thoughts were perceived by clients and clinicians to be crucial components connected with positive outcomes.

**Conclusions:**

The acceptability of CBT-f-DDD to both clients who completed therapy and clinicians was somewhat supported. The treatment was also deemed conditionally feasible for wider implementation. It is recommended that CBT-f-DDD be further refined in line with client needs, perceived mechanisms of change, professional training needs, and organisational factors.

**Trial registration:**

This study was embedded within a trial registered with the ISRCTN (registration number: ISRCTN97686121; registration date: 5th January 2023; 10.1186/ISRCTN97686121).

**Supplementary Information:**

The online version contains supplementary material available at 10.1186/s12888-026-08052-7.

## Background

Depersonalisation-derealisation disorder (DDD) is a psychological condition characterised by enduring experiences of dissociation leading to significant distress and impact on functioning [1, 2]. Depersonalisation involves the experience of disconnection from internal states [3], whereas derealisation is defined as a sense of detachment from one’s environment [4]. DDD is distinguishable from dissociation as part of post-traumatic stress disorder (PTSD) in the pervasive presence of dissociation above and beyond re-experiencing episodes in PTSD [2]. Furthermore, while PTSD stems from at least one traumatic event, DDD does not necessarily result from trauma [5]. Instead, DDD can involve triggers such as anxiety, substance use, low mood, and other mental health difficulties [6].

The prevalence of DDD is approximately 1% in the general public [7]. However, the condition remains overlooked in healthcare [8, 9], potentially due to dissociation often not being included within clinical training programmes, resulting in clinician difficulties in identifying relevant symptoms [10]. The journey to receiving a DDD diagnosis can last seven to 12 years [11, 12]. Clients with DDD often describe challenges in explaining their highly subjective symptoms, experiences of loneliness, and feeling unheard when pursuing psychological therapy [13]. There are currently no National Institute for Health and Care Excellence (NICE) guidelines addressing the treatment of DDD. Pathways to accessing mental health treatment can be complex [14]; patients may, for instance, be referred through a series of different services without being offered suitable treatment [13]. The availability of appropriate psychological care that targets symptoms remains low, with only one national specialist DDD service within the National Health Service (NHS) in the United Kingdom (UK) [15].

A range of therapeutic treatment methods have been found to provide some improvements to symptoms. For instance, dialectical behaviour therapy focuses on emotional regulation and reducing harmful behaviours using mindfulness and acceptance approaches [16]. Recent research has focused particularly on cognitive behavioural therapy (CBT), with CBT studies demonstrating more robust evidence compared to other therapeutic modalities [17]. From a cognitive behavioural perspective, treatment for depersonalisation and derealisation addresses catastrophic cognitions and associated maintaining emotional, behavioural and physiological patterns [18]. Key components of CBT for DDD (CBT-f-DDD) include engagement and psychoeducation about DDD, developing the shared formulation, cognitive strategies, emotional regulation strategies, behavioural interventions, working with common co-morbid conditions triggering DDD, working with issues related to onset, working with predisposing factors, and staying well plans [19]. Significant improvements in symptoms were found in two CBT for DDD (CBT-f-DDD) clinical audits conducted within the NHS national specialist DDD Clinic [20, 21]. Secondary improvements in depression and anxiety were also noted in both studies, along with increased functioning in the earlier study.

Positive outcomes in CBT for other mental health difficulties have been linked to various components and processes. These include gaining a comprehensive understanding of difficulties, forging a strong therapeutic alliance [22–24], completing therapy in full, and having a reliable and non-judgmental therapist [25, 26]. Cognitive interventions also contribute to improvements in mental health difficulties through generating adaptive perspectives of distressing experiences [27, 28]. However, to date, no studies have examined the perceived therapeutic components that potentially influence DDD outcomes. Similarly, while CBT has generally been found to be acceptable and feasible across a range of clinical presentations and settings [29–31], this has not been investigated to date in those with DDD. A qualitative approach enables exploration of various aspects of acceptability [32] and feasibility embedded within broader accounts of how treatment is experienced.

The present qualitative study was embedded within a CBT-f-DDD feasibility randomised controlled trial (RCT) [33], the findings of which are reported separately [19]. The aims of the current study were to examine the potential acceptability of treatment to clients and delivering clinicians; investigate the feasibility of wider NHS provision; and explore clients’ and clinicians’ views on the potential components or processes within CBT-f-DDD that might have catalysed change in clients’ difficulties. Two research questions were developed:

1. To what extent do the experiences of clients and clinicians support the acceptability and feasibility of CBT for DDD?
2. What do the experiences of clients and clinicians suggest about potential underlying processes and components of CBT for DDD that may contribute towards treatment outcomes?

## Methods

Methods are reported in line with established criteria for qualitative research [34].

### Design and participants

In the CBT-f-DDD feasibility RCT, clients were recruited through London-based mental health services. All screened clients met the DSM-V diagnostic criteria for DDD [1] and were randomised to the treatment arm to receive 12–24 CBT-f-DDD sessions over six months, or the control arm to receive treatment as usual. The RCT protocol is presented separately [33], which also details the process of excluding clients experiencing dissociation in the context of primary PTSD.

Participants of the present study were recruited from the CBT-f-DDD feasibility RCT. Eligibility criteria were: for clients, allocation to the treatment arm, and completion of therapy and the final assessment; for clinicians, facilitation of full or partial therapy for at least one client. Clients who did not complete therapy were ineligible, as they were no longer considered active participants in the trial. Clinicians who provided partial therapy were included as sessions were terminated due to client disengagement and there were no indications that clinicians were withdrawing from participation.

### Procedure

Data was collected through semi-structured interviews. Interview schedules for clients (Appendix 1) and clinicians (Appendix 2) were drafted by the Trial Management Group, which included an expert by experience (JP). Questions for clients covered their overall experience of therapy, specific aspects that they found particularly important and useful, changes experienced during or after therapy, and their rapport with the clinician. Clinicians were asked about what worked well and less well in CBT-f-DDD, proposed changes to the therapy model, and considerations around providing the treatment more widely in the NHS.

Clients were approached after trial quantitative data collection was completed; clinicians were approached following the conclusion of work with allocated clients. Interviews were conducted by two researchers (CLMW and ND) either in-person or virtually through Microsoft Teams from January 2023 to January 2024, at which point all eligible participants had been recruited. All interviews were audio recorded, then transcribed and anonymised. Both interviewers wrote reflective summaries following each interview, documenting key points raised by participants and the interviewer’s reflexive notes. These summaries were shared with the research team to inform subsequent analysis.

### Analytic approach and theme development

Codebook thematic analysis (TA) was selected as the analytic approach as it offers flexibility and a primarily inductive approach, while also acknowledging a starting point focusing on particular topics. It is also well suited to a collaborative analytic approach and applied qualitative research topics [35, 36]. Analysis was conducted by first author CLMW with collaborative input and discussion with other team members throughout. Following data familiarisation through detailed reading of the transcripts and reflexive summaries, initial coding focused on a subset of transcripts that were broadly typical of general patterns of experiences across participants. These were also coded by other team members who brought varied expertise, including an expert by experience. Initial coding was discussed and revised collaboratively. The remaining transcripts were then coded, with iterative modifications to the structure and content of the codebook. Finally, the team provided further collaborative input into refining and naming themes to capture meaningful concepts, concerns and viewpoints expressed by interviewees.

### Research team positioning and reflexivity

The research team consisted of a trainee clinical psychologist specialising in CBT (CLMW), two clinical psychologists with CBT expertise (TN, EH) (one of whom (EH) was an expert in CBT-f-DDD and the Principal Investigator of the DDD trial), a research assistant in the DDD trial (ND), an expert by experience (JP), and a qualitative methodologist (NM). One author (TN) had no involvement in trial development and management, which helped reduce analytic proximity. Throughout the research, the team maintained a reflexive awareness of these positions and critically evaluated methodological processes including interview design and analysis.

The backgrounds of the team members were diverse and collaborative discussions were conducted at various stages of data analysis. Such collaboration mitigated risks in analysis by accommodating, amalgamating and negotiating the varied interpretations and theoretical allegiance held by different team members, ensuring appropriate balance.

## Results

### Participant characteristics

**Fig. 1:**
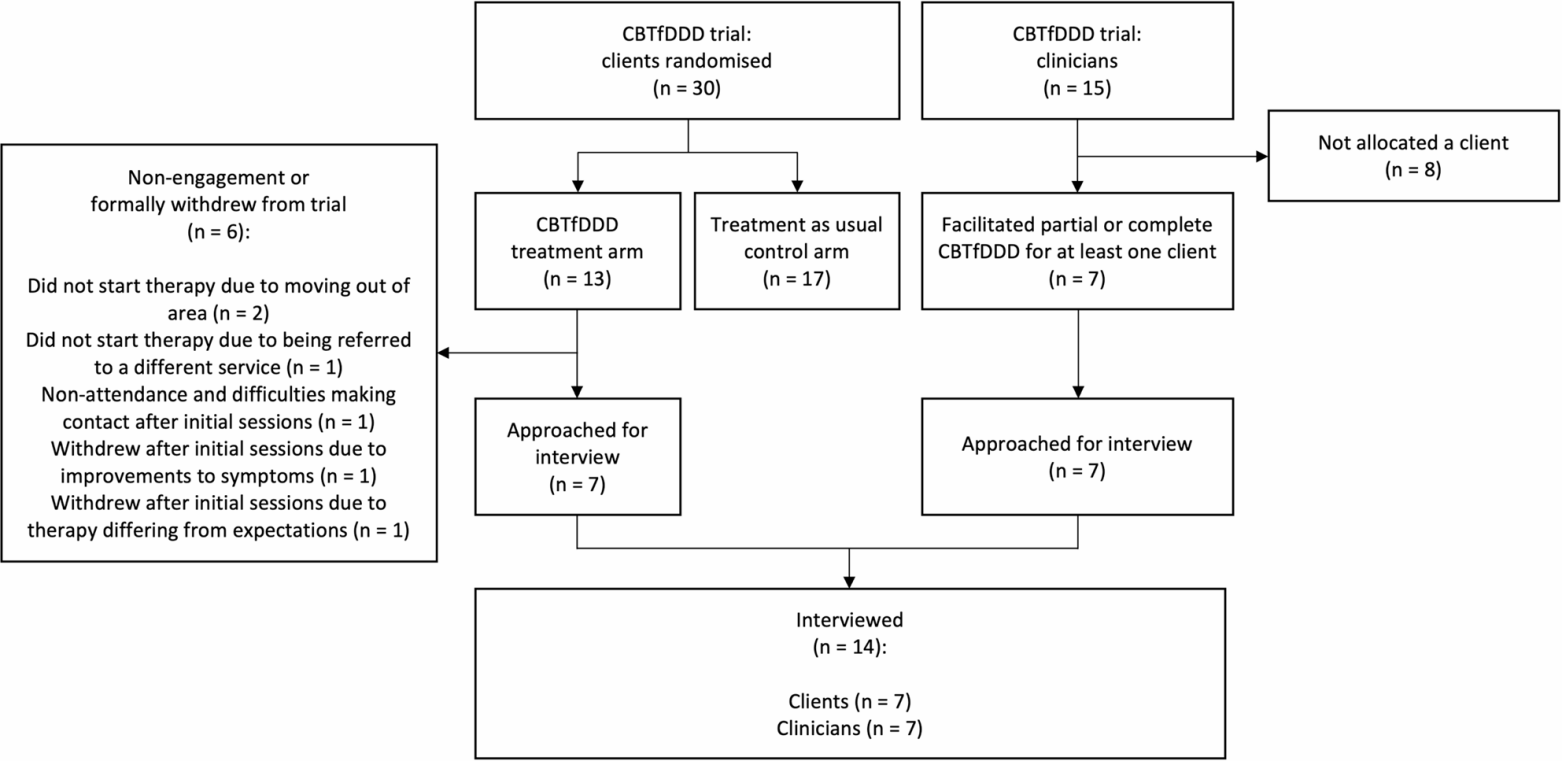
Participant flow diagram

A total of 14 participants were interviewed, comprising of seven clients who completed CBT-f-DDD and seven clinicians, all of whom were from NHS Talking Therapies for Anxiety and Depression (TTad) services, formerly known as Increasing Access to Psychological Therapies (IAPT) services. The length of interviews ranged from 29 to 54 min. The flow of participant recruitment is detailed in Fig. 1.

Table 1 present the characteristics of the interviewed clients and clinicians respectively.

**Table 1:** Client and clinician characteristics

Characteristic	Clients		Clinicians	
	*n*	%	*n*	%
Age				
< 20	1	14.3	0	0
20–29	4	57.1	1	14.3
30–39	2	28.6	3	42.9
40–49	0	0	3	42.9
Gender				
Female	4	57.1	6	85.7
Male	3	42.9	1	14.3
Ethnicity				
White British	6	85.7	3	42.9
White Other	1	14.3	2	28.6
Black African	0	0	1	14.3
Black British	0	0	1	14.3
Employment status				
Employed	4	57.1	N/A	N/A
Student	3	42.9	N/A	N/A
Role				
CBT Therapist	N/A	N/A	6	85.7
Clinical Psychologist	N/A	N/A	1	14.3
Mode of therapy sessions				
Mainly or fully online	6	85.7	N/A	N/A
Mainly or fully in-person	1	14.3	N/A	N/A
Clients seen in trial				
3	N/A	N/A	1	14.3
2	N/A	N/A	1	14.3
1	N/A	N/A	5	71.4

All interviewed clients were therapy completers – two clients attended 17 sessions; others attended 12, 16, 18, 19 and 21 sessions respectively. In addition to DDD as the primary difficulty, six clients experienced comorbid mental health difficulties – three had generalised anxiety disorder (GAD), two had GAD and depression, and one had depression. The mean age of DDD onset was 18.57 years (SD = 7.55) and the mean duration of DDD was 10.43 years (SD = 8.28).

Two clinicians had been qualified for nine years, while others had each been qualified for one, two, seven, 10 and 13 years respectively. Four clinicians completed therapy with their allocated clients. Three clinicians, all allocated one client, facilitated two to four sessions before their client withdrew.

### Overview and presentation of themes

Themes and sub-themes were organised in a hierarchy and are presented to broadly follow the chronology of clients’ journeys through therapy (see Table 2). Theme 4, treatment implementation in the NHS, was based solely on clinicians as clients were not interviewed about this topic. Quotes by clients are indicated by a number identifier, whereas therapist quotes are denoted by a “T” followed by a number identifier.

**Table 2:** List of themes and sub-themes

Themes	Sub-themes and third order themes
Reception of therapy	Accessing a DDD-targeted therapy
	A CBT approach
	Seeing changes to symptoms and the relationship with DDD
	Considering mental health difficulties other than DDD
Possible therapeutic processes as drivers for change	Building therapeutic rapport
	Tailoring therapy to clients
	Constructing a shared understanding of DDD
	Using targeted therapeutic techniques
Improving the therapy	Arranging sessions to maximise engagement
	Managing beginnings and endings of therapy
	Retaining therapy discussions
Treatment implementation in the NHS	Sense of familiarity in facilitating therapy
	Forming effective treatment pathways
	Supporting training and supervision for clinicians

### Theme 1: reception of therapy

This theme captured the experiences of clients who completed therapy and clinicians engaging in CBT-f-DDD and its effects.

#### Accessing a DDD-targeted therapy

Several clients described negative experiences of accessing support and feeling unseen before CBT-f-DDD, citing a lack of DDD awareness among professionals as the key reason their needs were not addressed adequately. This was compounded by having no one to turn to and needing to find answers about symptoms themselves. Noting the scarcity of psychological interventions specifically for DDD, some appreciated the opportunity to access a targeted treatment and have the gravity and consequences of their symptoms recognised. Clients expressed a sense of relief due to being validated and heard for the first time, in contrast with past help-seeking experiences.

I’m very thankful that I could be a part of this … there are too many people like me who are struggling. And we are, like, normal, functioning people … it’s, like, this horrible thing (DDD) and no one will ever tell you what’s going on with you. So I think it’s a really important job you’re doing because you can change a lot of lives. (012)

While speaking about difficulties in a new setting was daunting, clients ultimately found therapy to be rewarding. Two clients described a highly positive therapy experience (012, 019), three (005, 009, 011) mostly favoured the treatment, and two (001, 025) considered CBT-f-DDD to be broadly helpful but noted some reservations.

All clinicians held CBT-f-DDD in positive regard. Most clinicians also considered the therapy to be well-accepted by their clients. Some clinicians saw favourable therapy outcomes and stronger engagement as indications that clients found the treatment satisfactory.

#### A CBT approach

Clients generally considered CBT appropriate for treating DDD as it specifically targeted processes that perpetuate DDD with subsequent impact on symptoms. They highlighted the structured and rational approach of CBT as a useful way to navigate difficulties. However, one client thought that greater examination of the origins of symptoms would have helped them with understanding DDD.

Being able to talk to someone who can help you break it down and look at things a little bit more logically is definitely a big help. (019)

Clinicians’ views mostly accorded with clients’ regarding the appropriateness of a CBT approach to DDD, although those whose clients did not complete therapy found it more challenging to gauge whether the treatment model resonated with their clients.

#### Seeing changes to symptoms and the relationship to DDD

Six clients described improvements in DDD. One client experienced a more tangible sense of reality, which was absent before therapy. Clients generally did not expect the degree of improvement experienced, due to a pre-established sense of hopelessness and symptoms previously perceived as somewhat immovable.

My symptoms were atrocious before I started … I had no idea that I was ever going to be able to get below a certain baseline of being chronically dissociative or derealised … since I’ve had this therapy I keep getting little peaks of as non-dissociated as I think I can be, which is massive. (005)

Clients perceived that the therapy improved their relationship with DDD. They felt equipped with an increased sense of agency and self-belief in managing symptoms, including the client who experienced little changes to symptoms. Moreover, clients saw DDD as addressable and malleable following therapy, discovering a newfound sense of hopefulness. DDD was no longer seen as a condition that wielded significant power over them and engaging in daily living became more achievable.

It feels much more manageable. And I now know it’s (dissociation) not permanent … it’s in my control, and that it will go away … and it’s not scary anymore, because I know that it can change. (009)

#### Considering mental health difficulties other than DDD

During therapy, two clients felt unsure that DDD was their primary difficulty. While both deemed the sessions to be useful overall, the process of identifying DDD in the first place felt confusing. One client had constructive discussions with their clinician about differential diagnoses, while the other felt frustrated that their queries were not responded to clearly.

I can see how this (DDD) would fit if you looked at it this way, but could you interpret this set of symptoms in other ways? (001)

A handful of clinicians discussed that CBT-f-DDD was not as suitable for individuals with comorbid difficulties that could have been an alternative focus for treatment. A few clinicians facilitated discrete interventions to target non-DDD difficulties deemed important to the clients’ broader needs, such as low mood and anxiety. Clinicians found it challenging to conceptualise experiences encompassing a range of complex difficulties and felt lost with how to navigate sessions.

What I found tricky was trying to make sense of everything that was going on for them … I was jumping from one thing to another, but I was really trying at the same time to stick to one thing to work on with them. (T4)

### Theme 2: possible therapeutic processes as drivers for change

This theme focuses on aspects of therapy that clients and clinicians perceived to have facilitated or hindered change for the client. Change was conceptualised broadly and not defined solely as improvements in symptoms.

### Building therapeutic rapport

Clients valued clinicians being attentive, friendly and empathic to form a strong therapeutic alliance, appreciating that their experiences were validated, normalised, and believed by clinicians as DDD had often felt overlooked in the past.

I felt like I was actually being cared for. It just felt genuine … It makes a massive difference … just the little attentions to detail. (005)

A few clients felt slightly distant from their clinicians, adding that developing therapeutic alliances individually might have been challenging due to high clinician caseloads. This was considered a pity but did not substantially impact the quality of therapy.

Their experience of my DDD was sort of a job and my experience of my DDD is sort of a pretty disastrous, horrendous experience … that’s no bad thing they’re doing- This is their vocation. But I could certainly tell that I was one of a number that they saw in a week. (025)

Clinicians generally felt that clients with stronger therapy engagement saw more favourable outcomes. Good rapport also contributed to a sense of safety, which helped clients engage in discussion about difficulties and try therapeutic techniques. Clinicians who worked with clients that withdrew found it challenging to develop rapport, noting that clients appeared uncomfortable with sharing their experiences, potentially due to anxiety.

#### Tailoring therapy to clients

Some clients appreciated adjustments clinicians made according to their needs, such as co-producing the therapy which allowed discussions to be firmly rooted in their subjective experiences.

It never felt like [clinician] was looking for specific answers or anything. It was more just like me leading the sessions which was nice. (011)

Clinicians described that working at clients’ pace afforded sufficient time for clients to absorb discussions from sessions, which was particularly useful for half of the clients who experienced memory issues as part of DDD. Some clinicians also facilitated more frequent recaps and encouraged notetaking, which clients found constructive.

#### Constructing a shared understanding of DDD

Some clinicians discussed that tracing the origins of DDD helped to reassure that symptoms do not randomly emerge. Both clinicians and clients thought that collaboratively conceptualising the processes behind DDD symptoms provided a roadmap for treatment. Clients valued clinicians’ specialist DDD knowledge, which helped reframe negative preconceptions about DDD. Notably, one client highlighted that their clinician’s insight helped them realise that dissociation deprived them of experiencing emotions fully.

At the start of the sessions, there definitely was a part of me that thought actually sometimes it (DDD) can be a good thing for me. I think having that constant explanation of why I was dissociating was useful and how actually it wasn’t serving me because I was blocking out positive emotions as well as negative. (011)

Some clinicians found diary keeping key for gaining a thorough understanding of clients’ dissociation and its impact. However, clients’ views were mixed. Some discussed that this helped them identify patterns: *“We found out that my DP (depersonalisation) was connected to stress … stress caused anxiety and then anxiety caused DP”* (012). Others stated that diary keeping did not produce meaningful interpretations: *“There’s no emotional information there. It’s just a step-by-step map of things I’ve done”* (001).

#### Using targeted therapeutic techniques

This sub-theme encapsulated the cognitive and behavioural techniques, specific to clients’ subjective dissociative experiences, that were perceived to help alleviate distress.

##### **Reframing thoughts and beliefs**

Around half of clients found that rationally reframing DDD-related beliefs (e.g.: *“My life is going to become significantly harder”* (025)) reduced the power of the thoughts and the fear of DDD. Several clinicians noted that shifting clients’ negative beliefs was also important preparation for further interventions. Clients and clinicians shared that evaluating and challenging unhelpful principles and assumptions, “rules of life”, about perfectionism indirectly reduced dissociation, as clients imposed substantial pressure on themselves that maintained anxiety and ultimately DDD. For instance, one client initially adhered to the rule of *“I have to do everything 100% to be successful”* (012), then realised *“I can now put 75% to things or less … I would put less pressure on myself and*,* therefore*,* there is less stress”* (012).

##### Testing predictions

Clients tested out the negative predicted outcomes in real life situations, the impact of deviating from their “rules of life”, and different ways of coping. Common learning points included the processes that worsened and improved their symptoms, ways of living with DDD, and understanding that the consequences of DDD were not as adverse as expected. Clinicians stated that these takeaways helped clients change their behaviours to manage DDD more effectively.

##### Exploring grounding and coping strategies

A few clients realised that sitting with uncertainty was more sustainable in accommodating dissociation than avoiding thinking about their symptoms. Most clients elaborated that grounding strategies shifted attention from dissociative sensations to other stimuli, reinforcing a sense of reality and enabling them to stay rooted in their surroundings.

Dissociation is like being blind to time and being blind to place. But using the grounding techniques is almost a threat to that. I’d have to actively step out of this river that I can’t get out of and then stand on the bank and look at the river. (009).

A handful of clients described that grounding did not produce meaningful effects following initial attempts, leading to reduced motivation to continue practicing. Those who found it challenging to practice techniques for homework also cited memory issues and the busy nature of their occupation.

### Theme 3: improving the therapy

This theme covers issues and suggested changes regarding the practical and conceptual aspects of treatment to better attend to client needs.

#### Arranging sessions to maximise engagement

Clinicians and clients both proposed that weekly sessions with minimal breaks would help with integrating therapeutic techniques into daily life and maintaining accountability. Some had long breaks between sessions, which impeded progress.

The momentum is really, really important. There were a few periods of time … I couldn’t attend therapy for a few weeks, and they were away … it felt like we were just spending sessions playing catch-up. (005)

A few clients and clinicians raised that online sessions hindered engagement, with a couple of clinicians suggesting that this format might have increased dissociation for clients. Conversely, one client who had online sessions did not experience increased symptoms and considered convenience as an advantage.

#### Managing beginnings and endings of therapy

Some clients stated that being provided an expectation about what therapy might look like, especially if they had not engaged in CBT before, would have helped ease uncertainties. Furthermore, treatment goals felt unclear which led to some feeling puzzled about what the work might built toward. A handful of clients discussed that too many initial sessions were spent discussing CBT theory, where some sessions could have been repurposed to introducing coping strategies. This originated from a desire to gain immediate relief to distressing symptoms, which were often heightened at the start of therapy.

Clients found therapy conclusions to be negative. Most described feeling blindsided by how swiftly endings arrived, with insufficient time and readiness to wrap up. Clients proposed that having more reminders from clinicians would have cultivated more contained conclusions. Two clients further felt that the post-treatment plan was confusing, noting difficulties in navigating healthcare processes to access support again if needed.

At the end, is it like, I’m discharged, and if something happened in future where I felt like I needed more help, I would start from scratch on the whole process? (001)

#### Retaining therapy discussions

Approximately half of the clients conveyed difficulties holding onto therapy discussions. One client related this to memory issues as part of their DDD. Despite a few maintaining their own therapy notebook throughout sessions, clients were keen for clinician input on notes and suggested collaborative note-taking – this approach was utilised by one clinician.

So I would have a copy (of notes), you would have a copy, we’d go through it together … “What are you writing down so I can write down the same?” So it felt like an interaction rather than, like, something stale … I think that’s what brought it to life. (T15)

#### Theme 4: treatment implementation in the NHS

Deriving solely from clinician interviews, this theme focused on views on the processes associated with implementing CBT-f-DDD more widely in the NHS, including relevant opportunities and barriers.

#### Sense of familiarity in facilitating therapy

All clinicians expressed that their pre-existing CBT knowledge and skills were transferable to DDD, which made facilitating the therapy feel comfortable and familiar. The three clinicians that worked with clients who did not finish treatment were only able to speak to the initial treatment stages, namely assessment, providing psychoeducation, and constructing a formulation. While the aforementioned sense of familiarity still helped, they also wished they could have completed therapy to fully assess the utility of CBT-f-DDD.

#### Forming effective treatment pathways

All clinicians saw a clear need to create appropriate treatment pathways for CBT-f-DDD within public health services. Clinicians highlighted that the remit of services should be well-defined, as this would ensure that the full range of DDD severity is covered and reduce the number of service users falling through provision gaps.

I think it seems perfectly feasible. As with any provision it has to be thought about what’s primary care, what’s secondary care, what’s the pathway … how many people are we going to get through the door … what if we (TTad) start getting people with more severe- I’m not really sure there’ll be anywhere we could send them. (T13)

Most clinicians deemed CBT-f-DDD appropriate and viable to be delivered in TTad. They highlighted the advantage that clients could self-refer to TTad without needing to navigate complex referral processes for secondary or tertiary services. Noting the remit of TTad, some felt that primary care might be most suitable for individuals likely to benefit from short-term work.

It might be a case that the end goal (for treatment in TTad) is not to completely get rid of everything DDD symptom wise and just to make a starting point for improvement that they can continue with working on independently. (T9)

#### Supporting training and supervision for clinicians

Roughly half of the clinicians thought current knowledge of DDD among professionals was lacking in terms of both understanding and treating it and that specialist training and supervision could address this knowledge gap. This was considered a prerequisite for rolling out CBT-f-DDD in the NHS. Most stated that DDD teaching would be best embedded into core therapy training, as this would equip all future therapists consistently. However, some acknowledged that it might be challenging to add DDD into an already full curriculum. Service level training was also proposed to reach qualified clinicians and maximise continuity.

There’s so much staff turnover (in TTad), you can’t guarantee that if you did a training to every borough, you’re going to capture everyone. Whereas I think within those courses (core CBT training), you can reach a wider net. (T1)

Quality supervision was also deemed crucial towards supporting future clinicians who would deliver CBT-f-DDD. Some proposed hiring a specialist supervisor externally, though recognised the limited number of experts in the field. Others suggested training appropriate staff within services to become supervisors, though a few clinicians shared queries around this process.

At what point would that person become someone who could supervise a DDD case? Would they need a little bit more training in it? Would the half day we’ve already had (trial training) and a couple of cases be enough? Probably not. (T13)

## Discussion

### Acceptability and feasibility of CBT-f-DDD

#### Acceptability of the treatment

Clients who completed therapy and clinicians generally had a positive experience of CBT-f-DDD and considered the treatment as suitable for DDD. Strong therapeutic alliances appeared to cultivate a sense of safety, leading to a willingness to engage in new ways of thinking and acting as part of the therapy. However, those who felt unsure about their primary DDD diagnosis saw relatively less utility to the treatment, echoing the disorder-specific nature of CBT protocols more broadly [37]. Clinicians might have found working with comorbidities challenging due to usually treating one condition at a time in TTad services. Clients shared their negative journeys to DDD diagnosis and treatment and feelings of invalidation before the trial [13, 14], so the opportunity to access specific CBT-f-DDD helped engagement in the treatment [38].

Improvements in DDD symptoms and functioning mirror quantitative findings [20, 21], with DDD described as more manageable and less feared, probably due to the reduced belief in these symptoms being dangerous [18]. Although some clients reported transient distress in sessions, this was likely due to discussing difficulties in detail rather than using avoidance strategies [39].

Attitudes toward the beginnings and endings of therapy were broadly negative. Not providing an expectation of therapy could have impacted clients’ view of CBT-f-DDD before sessions [40]. Moreover, disorganised endings might be difficult as this could weaken containment [41, 42]. If therapy concluded sooner than anticipated, this might lead to worries about reverting to coping with DDD alone [13], which particularly relates to the context of frustrating past experiences of seeking DDD treatment. Clients might have also found endings challenging as treatment pathways for dissociation within current NHS services are unclear [43], potentially leading to a sense of confusion upon completing CBT-f-DDD.

It should be noted that interpretations relating to acceptability cannot be extrapolated to therapy non-completers as they were not interviewed. Among the six non-completers, two did not start therapy due to moving out of area and one did not start therapy due to being referred to a different service – these three clients would have been unsuitable to interview as they did not experience any part of the therapy. The remaining three clients were withdrawn from the trial and no follow-up contact attempts were successful – one client stated an improvement in symptoms as the withdrawal reason and another reported that therapy differed from expectations, while the final client was deemed withdrawn due to continued absence from sessions and being uncontactable. Therefore, the latter two clients might have had a more negative view of CBT-f-DDD, whereas the first client likely found the therapy positive.

#### Feasibility of implementing the treatment in the NHS

Clinicians viewed TTad as a feasible setting to start treatment implementation in the NHS, so long as pathways are sculpted according to local needs. This mirrors the stepped care model of therapies for depression and anxiety applied across the UK [44, 45]. There may also be limits to feasibility however, as stricter policies in TTad routine practice compared to the trial could lead to earlier, unplanned discharge due to non-attendance.

Requiring appropriate training and supervision for clinicians as a prerequisite for NHS implementation could prevent public health ramifications [46]. Incorporating DDD into core CBT training could support feasibility in the long run. Based on the sense of comfort reported by therapists from the present study in using CBT-f-DDD, others already qualified in CBT may also find they are able to add this specific treatment model to their existing repertoire confidently following appropriate top-up training. However, sourcing relevant expert supervision could prove challenging due to the scarcity of DDD specialists [14].

### Underlying CBT-f-DDD processes and components that potentially contributed to treatment outcomes

Having a strong therapeutic relationship might have motivated session engagement and in turn strengthened DDD outcomes [47]. As DDD symptoms can be challenging for others to comprehend [48], the use of a DDD-specific model likely facilitated collaboration between clients and clinicians in conceptualising DDD while remaining grounded in idiosyncratic lived experiences. For the few clients who felt distant from their clinicians, this may indicate that clinicians did not fully grasp clients’ subjective experiences. Issues with rapport building could have also arisen from sessions being online, as the computer screen might have acted as an invisible wall and led to derealisation [49].

Clients were facilitated to build an awareness that dissociation could involve strange and intolerable experiences but were not inherently life-threatening [18, 20]. Evaluating predictions through experiments potentially helped decrease catastrophising and fear around the nature and consequences of DDD [50]. Furthermore, using experiments to challenge rules of life related to perfectionism might have indirectly contributed to improvements in symptoms [48].

### Strengths and limitations

This was the first study to qualitatively explore experiences of CBT-f-DDD, incorporating the perspectives of clients and clinicians. All clients and clinicians who took part in the trial and were eligible for this study agreed to be interviewed. Interviewed clinicians ranged from recently qualified to very experienced. A team approach was taken throughout the research process, including conceptualising the study, drafting the interview schedule, discussing approaches to conducting interviews, data analysis and interpretation, and write-up. This allowed for reflexivity to be embedded into all parts of the study, as well as enabled various perspectives, including lived experience, in interpreting the data.

However, this study was small (seven clients and seven clinicians), constrained by the number of eligible participants within the treatment arm of the trial. Hence, it was challenging to discern whether views expressed by one or two participants were idiosyncratic and isolated, or representative of a more reliable and widespread pattern. The generalisability of the data was also hindered by the ethnically homogenous client sample, with all being White, whereas the control arm of the trial had greater ethnic diversity.

The lack of interviews with clients who did not complete CBT-f-DDD may be one reason for the generally positive accounts of therapy in our data, as some clients who dropped out of therapy may have held more critical views of CBT-f-DDD. Should contact have been successful, it would have been valuable to interview these clients to capture a wider spectrum of experiences. Our interpretations can therefore only be applied to individuals who completed CBT-f-DDD. Future studies should embed more robust processes to capture the views of clients who did not complete therapy, such as more explicitly stating upon recruitment the request for clients to engage in an interview even if therapy was not completed, as well as providing remuneration as an incentive.

The decision to interview clinicians who only delivered a small number of sessions before client withdrawal had disadvantages and advantages. For these clinicians, the therapeutic processes deemed more important might be naturally skewed towards the earlier stages of CBT-f-DDD. On the other hand, however, these clinicians commented on aspects of therapy content, delivery and engagement that might be associated with their clients withdrawing from sessions. In the absence of interviews with clients who withdrew, this offered unique perspectives that differed from the majority of the dataset.

The trial did not screen for personality disorders as participants were recruited from NHS services that would have identified these diagnoses through routine clinical practice. More active screening and exclusion processes for personality disorders for this study would have enhanced the robustness of sampling.

### Clinical implications

While CBT-f-DDD was considered tentatively acceptable by therapy completers and clinicians, further development of the therapy will help best meet client needs. CBT-f-DDD may not be as suited to individuals who report non-DDD comorbidities as their primary treatment goal. A key finding of this study was that clients experienced help-seeking negatively prior to the trial. It may be beneficial to integrate narrative-focused components into CBT-f-DDD, enabling clients to elaborate on their pre-therapy journeys in an open-ended manner, which may aid rapport building and making sense of difficulties. Clinicians can achieve more contained beginnings and endings to therapy through setting clear expectations and boundaries at the start, responding to clients’ queries about uncertainties in therapy arrangements, and prompting earlier discussions to collaborate on how to best conclude sessions. High session regularity would be beneficial and in-person therapy may be preferable over online sessions. Depending on clients’ difficulties and therapeutic trajectory, it may be appropriate to dedicate a larger proportion of time on cognitive interventions. It is also important to tailor interventions to clients collaboratively and navigate issues with homework engagement.

Additional resources, staffing and budget would required to accommodate CBT-f-DDD. It would be beneficial for NHS trusts to create “DDD Lead” roles that would spearhead and oversee these processes. Collectively, trust leads could form a national network for sharing knowledge and learning.

## Conclusions

This study was the first of its kind and analysed the experiences of clients who completed therapy and clinicians in a trial of CBT-f-DDD. The tentative acceptability of the treatment was illustrated by the positive reception to the treatment, therapeutic benefits, and appropriateness of the approach. However, interpretations are hindered by the lack of interviews with therapy non-completers. Outcomes should also be interpreted in the context of caveats regarding the content and delivery of the therapy described by some clients. Clinicians supported the feasibility of providing CBT-f-DDD in the NHS, conditional on certain prerequisites and considerations being fulfilled to ensure the safe and effective provision of the treatment. Clients who completed therapy, and clinicians, perceived particular treatment elements, ranging from DDD-specific to broader therapeutic processes, to catalyse favourable outcomes relevant to clients’ experiences of DDD. Future clinical practice would benefit from particular focus on developing CBT-f-DDD in response to client needs, building further understanding of perfectionism in DDD and mechanisms of change in CBT-f-DDD, and considering systemic arrangements needed to support treatment implementation.

## Lived experienced commentary by Joe Perkins (Unreal Charity)

Having lived with DDD myself for 18 years now, and through my involvement with the charity Unreal (supporting people with lived experience of DDD – www.unrealcharity.com), this qualitative study highlights some key issues that people struggling with the disorder often face when trying to understand their experiences, verbalise symptoms, and ultimately seek and engage in therapy.

The central characteristic of DDD is a profound sense of disorientating unreality. Despite *reality testing* being intact, it can feel intensely frightening for people to feel like their connection to the outside world, and themselves, is uncontrollably slipping away, with many worrying they are “*going crazy*” or experiencing the likes of psychosis, brain tumours, etc. This spiral is exacerbated when approaching mental health services who often have little – in some cases, zero – awareness of what they are describing. It comes as no surprise that this paper highlights both medical lack of awareness being a key barrier to meeting clients’ needs, but also that the eventual accessing of expert knowledge through the intervention became central to people understanding, accepting and reframing their experiences. Self-diagnosis of DDD is common and people often verbalise within our charity peer support groups that receiving *clinical validation* – simply feeling believed, heard and understood – is enormously impactful.

Another recurring theme from our groups is a want – rather, prerequisite – for authenticity and connection when engaging with services. Therapeutic rapport was identified in this study as an important factor in how acceptable clients found the intervention and, despite not necessarily impacting the *quality* of treatment from the therapist’s perspective, poor rapport was linked with both early dropout and less favourable therapeutic outcomes. In addition, we frequently hear from others with DDD that therapy delivered online can feel significantly harder to engage with than in-person. With virtual therapy now commonplace and its general efficacy proven, this could potentially be an idiosyncrasy of dissociative conditions requiring further investigation: screens being an additional tier of feeling distant and disconnected from the process; reducing clients’ ability to engage with therapy and risking exacerbating symptoms.

It is reassuring to see the need to personalise therapy and consider comorbidities when working with people experiencing DDD identified. The charity encounters so many people who have vastly different peripheral experiences and symptomologies beyond the core sense of unreality, with equally varied responses to interventions, medications and self-care techniques. For example, some people find meditation and mindfulness relieves their symptoms, whilst others describe “*being alone with my thoughts*” as problematic. Discussing the varied nature of grounding techniques and coping strategies, and tailoring therapy to individual clients, reflects what we hear directly from many living with this condition – and the frustrations of services trying to rigidly apply general therapeutic frameworks to their complex cases in standard healthcare.

More than any of that however, I really hope this paper becomes a much-needed stepping stone in improving communication between therapists and clients. Fighting – often for many years – to access specialist help for DDD can be exhausting and demoralising; and despite the 1% prevalence amongst the general public, many mental health services simply are not aware of what clients are describing when they express an ongoing sense of detachment from the world. Raising awareness, refining interventions, and finding better ways of forming a shared understanding are all imperative to improving the landscape of DDD, and I hope this paper will inspire future research – continuing the efforts to help reduce intense suffering for so many people the world over.

## Supplementary Information

Below is the link to the electronic supplementary material.

Supplementary Material 1

## Data Availability

The datasets generated and analysed during the current study are not publicly available to protect participant privacy, due to the potential of deductive identification.

## Abbreviations

CBT: Cognitive behavioural therapy
CBT-f-DDD: Cognitive behavioural therapy for depersonalisation-derealisation disorder
COREQ: The consolidated criteria for reporting qualitative research
DDD: Depersonalisation-derealisation disorder
GAD: Generalised anxiety disorder
IAPT: Increasing Access to Psychological Therapies (now known as TTad)
NHS: National Health Service
NICE: National Institute for Health and Care Excellence
PTSD: Post-traumatic stress disorder
RCT: Randomised controlled trial
TA: Thematic analysis
TTad: NHS Talking Therapies for Anxiety and Depression (formerly known as IAPT)
UK: United Kingdom
