# Supplementary material for: Exploring client and clinician experiences of cognitive behavioural therapy for depersonalisation-derealisation disorder (CBT-f-DDD)
Source: BMC Psychiatry. 2026 May 5;26:493. doi: 10.1186/s12888-026-08052-7 (PMC13312645; doi:10.1186/s12888-026-08052-7)
Supplement: Supplementary file 1 — Supplementary Material 1 [file 12888_2026_8052_MOESM1_ESM.docx]

**Appendix 1 – client interview schedule**

1. How was your experience of the treatment you received?
2. Were there any specific aspects or components of the treatment that you found particularly helpful/useful?
3. Were there any specific aspects or components of the treatment that didn’t seem useful?
4. Were there any specific aspects or components of the treatment that you found particularly challenging or difficult?
5. If you could make any changes to the treatment, what would these be?
6. How did you get on with the therapist/clinician?
7. Do you think the treatment has brought about any changes for you?
8. Is there anything else you’d like to tell me about your experiences with the study or treatment?

**Appendix 2 – clinician interview schedule**

1. How did you find actually delivering the therapy?
2. What did you feel works well or is particularly valuable about CBT-f-DDD?
3. What did you feel works less well about CBT-f-DDD?
4. Which aspects / components did you feel confident / comfortable delivering?
5. Were there any aspects / components of the therapy you found difficult or challenging to deliver?
6. Are there any aspects of the therapy you would want to change?
7. What are your overall thoughts on CBT-f-DDD therapy?
8. Can you envisage using CBT-f-DDD again in the future?
9. What are your views on the feasibility of CBT-f-DDD within the NHS?
10. Is there anything else you’d like to tell me about your experience with the study/therapy?
